# Supplementary material for: Assessment of visually guided reaching in prodromal Alzheimer’s disease: a cross-sectional study protocol
Source: BMJ Open. 2020 Jun 4;10(6):e035021. doi: 10.1136/bmjopen-2019-035021 (PMC7279656; doi:10.1136/bmjopen-2019-035021)
Supplement: Supplementary data [file bmjopen-2019-035021supp001.pdf]

The assessment of visually guided misreaching in prodromal Alzheimer's disease: study protocol

Site-specific information

Site 1: Edinburgh

Patient recruitment in Edinburgh will take place at the Anne Rowling Regenerative Neurology Clinic (NHS Lothian), through a team led by Dr. Suvankar Pal. Patients who fit the recruitment criteria will be identified through the Rowling CARE-register and provided an information sheet and a notification of interest form.

All testing (patient and control) takes place in the Human Movement Laboratory, Department of Psychology, The University of Edinburgh.

Site 2: Norfolk

Patient recruitment will take place in the Julian Hospital in Norwich (NHS Norfolk & Suffolk). A team of research nurses will identify suitable participants who will be provided an information sheet and a notification of interest form.

All testing takes place in the Vision and Action Laboratory, Department of Psychology, The University of East Anglia.
