# Supplementary material for: Assessment of visually guided reaching in prodromal Alzheimer’s disease: a cross-sectional study protocol
Source: BMJ Open. 2020 Jun 4;10(6):e035021. doi: 10.1136/bmjopen-2019-035021 (PMC7279656; doi:10.1136/bmjopen-2019-035021)
Supplement: Author's manuscript [file bmjopen-2019-035021.draft_revisions.pdf]

BMJ Open is committed to open peer review. As part of this commitment we make the peer review history of every article we publish publicly available.

When an article is published we post the peer reviewers' comments and the authors' responses online. We also post the versions of the paper that were used during peer review. These are the versions that the peer review comments apply to.

The versions of the paper that follow are the versions that were submitted during the peer review process. They are not the versions of record or the final published versions. They should not be cited or distributed as the published version of this manuscript.

BMJ Open is an open access journal and the full, final, typeset and author-corrected version of record of the manuscript is available on our site with no access controls, subscription charges or pay-per-view fees (http://bmjopen.bmj.com).

If you have any questions on BMJ Open's open peer review process please email info.bmjopen@bmj.com

The assessment of visually guided misreaching in prodromal Alzheimer's disease: study protocol

Journal:	*BMJ Open*
Manuscript ID	bmjopen-2019-035021
Article Type:	Protocol
Date Submitted by the Author:	16-Oct-2019
Complete List of Authors:	Mitchell, Alexandra; The University of Edinburgh, School of Psychology, Philosophy & Language Sciences<br>McIntosh, Robert; The University of Edinburgh, School of Psychology, Philosophy & Language Sciences<br>Rossit, Stephanie; University of East Anglia, School of Psychology<br>Hornberger, M; University of East Anglia, School of Medicine<br>Pal, Suvankar; The University of Edinburgh, Anne Rowling Regenerative Neurology Clinic, Centre for Clinical Brain Sciences
Keywords:	Dementia < NEUROLOGY, Adult neurology < NEUROLOGY, Neurophysiology < NEUROLOGY, NEUROPATHOLOGY

SCHOLARONE™
Manuscripts

I, the Submitting Author has the right to grant and does grant on behalf of all authors of the Work (as defined in the below author licence), an exclusive licence and/or a non-exclusive licence for contributions from authors who are: i) UK Crown employees; ii) where BMJ has agreed a CC-BY licence shall apply, and/or iii) in accordance with the terms applicable for US Federal Government officers or employees acting as part of their official duties; on a worldwide, perpetual, irrevocable, royalty-free basis to BMJ Publishing Group Ltd ("BMJ") its licensees and where the relevant Journal is co-owned by BMJ to the co-owners of the Journal, to publish the Work in this journal and any other BMJ products and to exploit all rights, as set out in our licence.

The Submitting Author accepts and understands that any supply made under these terms is made by BMJ to the Submitting Author unless you are acting as an employee on behalf of your employer or a postgraduate student of an affiliated institution which is paying any applicable article publishing charge ("APC") for Open Access articles. Where the Submitting Author wishes to make the Work available on an Open Access basis (and intends to pay the relevant APC), the terms of reuse of such Open Access shall be governed by a Creative Commons licence – details of these licences and which Creative Commons licence will apply to this Work are set out in our licence referred to above.

Other than as permitted in any relevant BMJ Author's Self Archiving Policies, I confirm this Work has not been accepted for publication elsewhere, is not being considered for publication elsewhere and does not duplicate material already published. I confirm all authors consent to publication of this Work and authorise the granting of this licence.

For peer review only

The assessment of visually guided misreaching in prodromal Alzheimer's disease: study protocol

Alexandra G. Mitchell[1], Robert D. McIntosh[1], Stephanie Rossit[2], Michael Hornberger[3,4] & Suvankar Pal[5]

[1] School of Psychology, Philosophy & Language Sciences, University Of Edinburgh, Edinburgh, UK

[2] School of Psychology, University of East Anglia, Norwich UK

[3] Department of Medicine, Norwich Medical School, Univeristy of East Anglia, Norwich, UK

[4] Norfolk and Suffolk NHS Foundation Trust, Norwich, UK

[5] Anne Rowling Regenerative Neurology Clinic, Centre for Clinical Brain Sciences, University of Edinburgh, Edinburgh, UK

Corresponding author:

Dr Alexandra G. Mitchell

7 George Square,

Edinburgh,

EH8 9JZ,

UK

alexandra.mitchell@ed.ac.uk

+44(0)131 651 3232

Word count: 3,593

For peer review only

1 Abstract

Introduction: Recent evidence has implicated the precuneus of the medial parietal lobe as one of the first brain areas to show pathological changes in Alzheimer's disease (AD). Damage to the precuneus through focal brain injury is associated with impaired visually guided reaching, particularly for objects in peripheral vision. This raises the hypothesis that peripheral misreaching may be detectable in patients with prodromal AD. The aim of this study is to assess the frequency and severity of peripheral misreaching in patients with mild cognitive impairment (MCI) and AD.

Methods and Analysis: Patients presenting with amnestic MCI, mild-to-moderate AD, and healthy older-adult controls will be tested (target N=24 per group). Peripheral misreaching will be assessed using two set ups: a tablet based task of lateral reaching, and motion-tracked radial reaching (in depth). There are two versions of each task, where participants can look directly at targets (free reaching), and when they must maintain central fixation (peripheral reaching). All tasks will be conducted first on their dominant and then their non-dominant side. For each combination of task and side, a peripheral misreaching index (PMI) is then calculated as the increase in absolute reaching error between free and peripheral reaching. Each patient will be classified as showing peripheral misreaching if their PMI is significantly abnormal, by comparison to control performance on either side of space. We will then test whether the frequency of peripheral misreaching exceeds the chance level in each patient group, and compare the overall severity of misreaching between groups.

Ethics and Dissemination: Ethical approval was provided by the NHS East of England, Cambridge Central Research Ethics Committee (REC 19/EE/0170).

Key words: Alzheimer's disease, cognitive impairment, visually guided action, peripheral reaching, optic ataxia

For peer review only

2 Article Summary

Strengths and Limitations of this study

- The first study to systematically assess visually guided reaching in patients with cognitive impairment
- Includes a simple tablet-based task (lateral reaching) that could be readily translated to clinical to assess the presence of peripheral misreaching
- Case-control statistical tests of deficit are inherently low-powered, subtle deficits of misreaching may not be detected at the level of individual patients

For peer review only

3 Introduction

The pathophysiological cascade that leads to Alzheimer's disease (AD) can begin 20 years before the onset of cognitive problems such as memory loss (1). Longitudinal modelling of these changes has highlighted the precuneus, in the medial superior parietal lobe, as one of the first regions to be affected in this wave of change (Gordon et al., 2018). Focal damage in and around this area is known to be associated with deficits of visually guided action (3). One example of such a condition is optic ataxia, an impairment of misreaching typically reflected in peripheral vision (4,5). Patients with optic ataxia often do not often complain of this symptom and it is rarely assessed in clinical settings, and it can therefore go undetected (6). The changes observed in the precuneus in prodromal AD, and the link between the precuneus and optic ataxia, raise the hypothesis that optic ataxic misreaching may be detectable in patients with prodromal AD.

3.1 Specific hypothesis

The hypothesis that peripheral misreaching is a feature of AD predicts that individual patients with AD, and possibly those with MCI, will show an abnormally large inflation of reaching errors when aiming for targets in peripheral vision, as compared with targets in free vision. At a group level, patients with AD, and possibly MCI, may show significantly greater peripheral misreaching than healthy controls.

For peer review only

4 Methods

4.1 Study setting

The study is a collaboration between clinicians and University staff at the University of Edinburgh (UoE) and University of East Anglia (UEA). Details of recruitment and site information can be found in the supplementary materials. Data collection for this study began on 03/10/2019 and 8/48 patients have taken part. Data for healthy controls have already been collected.

4.2 Participants

Patients with a diagnosis of amnestic MCI or typical (amnestic) mild-to-moderate AD will be invited to take part. Mild to moderate AD will be defined by a score of at least 50 in the most recent administration of Addenbrooke's Cognitive Examination (ACE-III, Mathuranath, Nestor, Berrios, Rakowicz, & Hodges, 2000) If there is no recorded ACE-III score, clinical opinion of patient's condition will be used to assess eligibility.

Older adults without any known neurological disorders will be tested as a healthy control (HC) group. To achieve our target of 24 full data-sets per group (*Section 5.3*), we plan to test up to 30 participants in each group, allowing for possible withdrawals.

4.2.1 Inclusion criteria

For all participant groups, the ability to give informed consent is the initial inclusion criterion. Additional inclusion criteria are then applied to each group.

Control group inclusion criteria:

- Aged 50 – 75[1]
- No reported neurological or neurodegenerative conditions

MCI group inclusion criteria:

- Aged 45 – 85
- Clinical diagnosis of MCI with an amnestic pattern of presentation

[1] NB. The age-range for controls is targeted at the expected age range for patients, but the allowable range of ages for patients is wider than this, in order not to restrict recruitment unnecessarily.

For peer review only

AD group inclusion criteria:

- Aged 45 – 85
- Clinical diagnosis of AD

4.2.2 Exclusion criteria

For all participant groups, the following exclusion criteria are applied:

- Significant difficulty communicating or understanding instructions in English
- Significant, uncorrected visual impairment (e.g., cataract, macular degeneration, scotoma, amblyopia, strabismus)
- Conditions that could interfere with smooth hand movements (e.g. ataxia, essential tremor, severe arthritis)
- Prior history of stroke or TIA

4.2.3 Public and Patient involvement

Patients with MCI or AD and their carers were involved in the early stages of planning and development. A focus group was held at the Anne Rowling Clinic in Edinburgh where patients and carers had the opportunity to try out prototypes of the tablet-based reaching task and provide feedback on task design. This feedback was used to optimise the final task for patient accessibility and clarity.

4.3 Tasks

Two different set-ups will be used to assess peripheral reaching: a tablet-based assessment of reaching in the frontoparallel plane (lateral reaching), and a motion-tracking assessment of reaching in radial depth (radial reaching). Participants will complete two versions of each reaching task: a version in which participants look directly at targets before reaching to them (free reaching); and a version where central fixation is maintained (peripheral reaching). The critical outcome is a measure of the inflation of absolute reaching error in peripheral reaching relative to free reaching.

Before testing, the participant's dominant writing hand is identified (by self-report). All tasks are completed first on the dominant side, using the dominant hand, followed by

For peer review only

the non-dominant side and hand. Lateral reaching is completed first, followed by radial reaching. All tasks are performed in the same order for all participants.

4.3.1 Lateral reaching tasks

i. Stimuli & Apparatus

Stimuli are presented on a HP Pavillion x360 touch screen (active display 310x175mm, resolution 1920x1080 pixels). Tasks are controlled by a custom program written in OpenSesame version 3.2.8 *Kafkesque Koffka* (8). Participants are seated 40cm away from the screen which is positioned with either the right edge of the screen aligned to their midline (left-sided reaching, Figure 1A) or the left edge (right-sided reaching, Figure 1B). A start box (white rectangle, 2x2°, 13.96x13.96mm) is drawn at the centre edge (right or left) of the screen, aligned to participant's midpoint. In some tasks (detailed below) a white fixation cross is present (1x1°, 6.98x6.98mm), located 34.9mm (5°) directly above the start box. Targets are white circles (diameter = 2°, 13.96mm) presented along radial spokes at 28, 33 and 38° to the left (Figure 1A) or right (Figure 1B) of fixation. The experimenter sits across the table and monitors eye movements directly.

ii. Free reaching

For the first block in the lateral reaching task participants are not required to fixate, therefore the fixation cross is absent.

Participants initiate a trial by pressing and holding down the start box, which disappears at touch. At this point they are may search the screen for a target. After a short delay (250-750ms, randomised 100ms intervals) a target appears at one of nine possible locations. As soon as the target appears, participants look at it and make one smooth reach to try to touch the target. The target remains on the screen until a touch is recorded at any location, then the target disappears and a short beep (100ms, 440Hz) is played. The validity of the trial is then coded by the experimenter using a keyboard; 'y' – valid trial, 'e' – the participant did not move their eyes to the target, 'v' – invalid trial, and the start box reappears to begin another trial.

For peer review only

If the experimenter presses ‘e’ or ‘v’ that trial is repeated until a valid trial is recorded. The block ends after a minimum of 27 valid trials (3 per target location), or after 50 valid and ‘no eye-movement’ trials.

iii. Visual detection

This is a simple check to confirm that the participant is capable of detecting the targets when presented in peripheral vision, to be allow for a meaningful test of peripheral reaching (*Section 4.3.1iv*).

Throughout each trial the participant must gaze at the fixation cross. They initiate the trial by pressing and holding down the start box, which disappears when touched. In order to aid the maintenance of fixation, the fixation cross cycles between white and red at the screen refresh rate (60Hz). After a short delay (250-750ms), a target can appear at one of the nine locations for one second, or no target appears. This is followed by a short beep to indicate the end of the trial. The participant must verbally report whether or not a target was seen in that interval. The experimenter records the response using the keyboard (‘y’ – yes, ‘n’ – no). If the participant makes an eye-movement, the experimenter presses ‘e’ and the trial is repeated. The block ends after 15 valid (no eye-movement) trials, one for each of the nine target locations, and six catch trials with no target.

To progress to the peripheral reaching task, participants are required to detect at least 6/9 targets and correctly rejects at least 3/6 catch trials. Otherwise, testing is discontinued on that side of space.

iv. Peripheral reaching

For peripheral reaching participants are required to gaze at the fixation cross throughout each trial. A trial begins by pressing and holding down the start box. When touched, the start box disappears and the fixation cross cycles between white and red (at a rate of 60Hz) until the trial ends. After a short delay (250-750ms) a target appears at one of nine locations. Whilst maintaining fixation, participants make one smooth reaching movement to try to touch the target. The target remains on the screen until a touch is recorded at any location, and a short beep is played once the target disappears.

For peer review only

The experimenter then records the validity of the trial; 'y' – valid, 'e' – participant moved their eyes away from fixation, 'v' – invalid trial.

Invalid ('e' or 'v') trials are repeated until a valid trial is recorded. The block ends after a minimum of 27 valid trials (3 per target location), or after 50 valid and 'eye-movement' trials.

4.3.2 Radial reaching tasks

i. Stimulus & Apparatus[2]

An infrared motion-tracking camera (Optotrak Certus, Northern Digital Inc) is used to track the reaching movement. Infra-red-emitting diodes (IREDs) are taped to the tip of the right and left index fingers of each participant to track the reach in each hand. The Optotrak samples the IRED's 3D position at 100Hz throughout each 2000ms trial. The task is controlled by custom software written in LabView (National Instruments) programming environment.

Participants are seated with their head placed in a chin-rest in line with the middle of the display. Stimuli are back-projected via a mirror onto a screen (1000mm wide x 750mm deep) that lies flat in-front of the participant. A webcam is placed on the screen 50cm directly in-front of the participant, as a fixation point. The live webcam image feeds into a separate laptop, allowing experimenter to monitor gaze. A start-button is aligned to the centre of the screen, positioned 10cm in-front of the participant, 40cm away from fixation (Figure 2). Targets are white circles (diameter = 1.60°, 13.96mm) presented at 4 eccentric locations (11.4, 22.6, 33.4 and 43.6° away from centre) on each side (Figure 2).

[2] The stimuli and apparatus reported here are specific to UoE. At the second site, UEA, motion tracking was performed a Qualisys AB system (Gothenburg, Sweden). Targets are green LEDs (diameter = 0.68°, 6mm). The experiment is presented on a grey table (100x100cm) and the experiment is run through Psychophysics Toolbox (11) in MATLAB (Mathworks, USA).

For peer review only

ii. Calibration

A calibration procedure is carried out before the reaching tasks to record the IRED position at the actual target location. A target is displayed at one target location and the participant is instructed to cover it completely with their reaching finger. Once the target is covered, the experimenter presses the start button and the finger location is recorded for 2000ms. A beep plays after 2000ms, indicating that the participant can move their hand away from the target position. Another target appears at the next location and the same procedure is repeated. Calibration is run using the ipsilateral hand for four targets on the left side, and four on the right.

iii. Free reaching

Participants initiate a trial by pressing and holding down the start button. As soon as they push the button down, participants may look around the screen for a target. After 250-750ms a target appears, participants then look directly at the target and reach to touch the target in one smooth movement. Optotrak recording is initiated simultaneously with target appearance, and the target disappearance is the simultaneous with the end of the recording after 2000ms. When the target disappears a short beep (100ms, 440Hz) plays, the participant leaves their finger at its landing position until they hear the beep. After the trial, the experimenter codes the trial validity with a key-press; 'Return' – valid, 'F1' – no eye-movement, 'Esc' – invalid trial. If an invalid trial ('F1' or 'Esc') is coded the trial gets recycled to the end of the shuffled trial.

The block ends once 28 valid trials (7 per target location) are recorded, or after 50 valid and 'no eye-movement' trials.

iv. Peripheral reaching

To assess reaching accuracy in the periphery participants are required to look directly at central fixation (the webcam) throughout each trial. Participants initiate a trial by pressing and holding down the start button. After 250-750ms a target appears. Whilst maintaining gaze on the webcam participants make one smooth reaching movement to try to touch the target. After the reach, participants leave their finger at its landing position until a short beep (100ms, 440Hz). The target remains on screen for 2000ms

For peer review only

after the trial begins. The motion-tracker records the reach throughout the 2000ms trial. At the end of the trial, the experimenter codes trial validity; ‘Return’ – valid trial, ‘F1’ – eye movement during trial, ‘Esc’ – invalid trial. If an invalid trial (‘F1, ‘Esc’) is recorded then the trial is recycled to the end of the shuffled trial list.

The block ends after 28 valid trials (7 per target location) are recorded, or after 50 valid and ‘eye-movement’ trials.

For peer review only

5 Analysis plan

5.1 Lateral reaching task

For the critical analyses, a single measure of *reaching accuracy* is taken for each participant, for each combination of viewing condition (free, peripheral) and side (non-dominant, dominant). For each response, the absolute error (in mm, x and y axis) is recorded as the distance of the reach endpoint from the target midpoint. The median absolute error is then calculated for each target eccentricity, across responses to the 3 targets at that eccentricity, for each combination of viewing condition and side. The average absolute error is then calculated as the mean of the medians for the 3 eccentricities, to give the single measure of reaching accuracy for each viewing condition at each side.

For the comparison of individual patients against control performance, the data are further compressed to a single index of performance per side, by subtracting reaching accuracy in the free vision condition from the peripheral condition. We call this value the *peripheral misreaching index (PMI)*.

5.1.1 Analysis of single case deficits

We will screen the control group for outliers that might suggest abnormalities, as such values would reduce the (already low, see Figure 3) power to detect single-case deficits. We will use a robust method of outlier detection based on the median absolute deviation (MAD). The MAD can be multiplied by the consistency constant 1.4826 to estimate the standard deviation, assuming a normal distribution. Each control participant's PMI can be expressed a modified Z-score (Z') by subtracting the group median, divided by the median absolute deviation *1.4826. If Z' exceeds 2.5, that participant will be excluded, and replaced. Our simulations suggest that, for a group size of 24, we would expect to exclude (on average) < 1 participant (~0.67) by this criterion.

We will next assess, for each side, whether the PMI of controls is related to age or sex, by computing Pearson's correlations. If the correlation is ≥ .3 on either side, then that variable will be included as a covariate in the subsequent case-control comparisons for both sides.

For peer review only

Case-control comparisons will then be run to compare each patient's PMI against control performance. These comparisons will be based upon Crawford & Howell's (1998) modified t-test; or, if covariates are included, we will use the updated test of deficit (Crawford, Garthwaite and Ryan, 2011). As we are testing for an increased PMI in each patient, the tests will be one-tailed, with alpha level set to .025, to constrain the expected Type I error rate to .05 across the two sides. If a patient shows a significant deficit on either side, they will be classed as showing peripheral misreaching. If a patient meets an unadjusted criterion for a deficit (.05), but not the adjusted criterion (.025), they will be classified as showing borderline peripheral misreaching.

Finally, a binomial test will test whether the rate of observed peripheral misreaching exceeds the rate expected by chance (i.e. the per-patient adjusted alpha level of .05). A significant outcome ($p < .05$) for either patient group will indicate that peripheral misreaching is a feature of this patient group. The observed rate of peripheral misreaching will provide an estimate of how common it is. We will run a further analysis including borderline cases, and compare the rate of peripheral misreaching in each patient group against the appropriate chance level of (i.e. the per-patient unadjusted alpha level of .10).

5.1.2 Group-level analysis

The case control approach will be complemented by a group-level ANOVA of reaching accuracy, as measured by the PMI, with the between-subject factor of group (HC, MCI, AD) and the within subject factor of side (non-dominant, dominant). This analysis will test whether the average severity of peripheral misreaching in each patient group significantly exceeds that observed in healthy controls.

5.1.3 Exploratory analyses

More detailed analyses will be run with a between subject factor of group and within subject factors of side, eccentricity and viewing condition. These analysis will be conducted using dependent variables of absolute reaching error, directional (signed) reaching error, reaction time and movement time. The expectation is that peripheral misreaching will manifest as a fixation-directed bias, which is exacerbated at higher eccentricities.

For peer review only

5.2 Radial reaching task

IRED speed is used to determine onset and offset of the reaching movement. Movement onset is defined as the first frame in which the IRED's speed exceeds 50mm/s (and maintains that speed for up to 100ms). Movement offset is defined as the first subsequent frame in which IRED speed falls below 50mm/s. The landing position of the movement is defined by the x and y coordinates in the final frame of the movement, and will be recorded as errors relative to true target locations recorded during calibration for each participant.

An initial analysis of PMI for the radial reaching task will be performed, restricted to the two most eccentric target positions (33.4 and 43.6°). Case-control comparisons follow the plan for the lateral reaching task (*Section 5.1.1*), to estimate the rates of peripheral misreaching, and borderline peripheral misreaching, in the two patient groups. Due to different experimental set ups between the two test sites (UoE, UEA), each patient will be referenced to the same-site control data for case-control comparisons.

A group level ANOVA of PMI, restricted to the two most eccentric target positions, will similarly follow the plan for lateral reaching (*Section 5.1.2*). We will include site (UoE, UEA) as an additional covariate. Subsequent, more detailed analyses, will also follow the plan for lateral reaching (*Section 5.1.2*) Since motion tracking also provides kinematic variables on reaching trajectories, we also aim to examine the dependent variables peak speed and time to peak speed, as well as average spatial trajectory of reach.

5.3 Power considerations

The target sample sizes (N=24 per group) are based on power considerations related to the main inferential analyses, the case-control comparisons, and binomial tests of rates of peripheral misreaching deficits for the lateral reaching task.

The control sample size of 24 will provide close to the maximum power for case-control tests of deficit (figure 3A). Note that high power for these comparisons is inherently unachievable unless the deficit being tested for is very large. We do not know how large any misreaching deficits may be in our patient groups, but our control sample size will

For peer review only

provide close to the maximum achievable power to detect them if they exist. Figure 3B illustrates more fully the relationship between deficit size (D) and power, for the adjusted alpha level (.025) and unadjusted alpha level (.05) by which we will determine peripheral misreaching deficits and borderline cases respectively (*Section 5.1.1*).

The main hypothesis is that peripheral misreaching will be found in a significant proportion of patients with AD and MCI. For one-sample binomial test to determine whether the observed rate of peripheral misreaching exceeds the chance level of .05, a sample size of 24 has > .9 power. Provided that the true population proportion is at least .2 (1 in 5). This is appropriate to our aims, since peripheral misreaching would be of limited significance in these clinical populations if its prevalence were less than 1 in 5.

For peer review only

6 Ethics and dissemination

This protocol was approved by UK Health Research Authority, by the East of England, Cambridge Central Research Ethics Committee on 13 June 2019 (REC reference 19/EE/0170).

All patients will provide informed consent, highlighting the voluntary nature of the study and their right to withdraw. If there is any doubt about the ability of the patient to provide informed consent, then this patient will not be recruited. There are no direct risks associated with taking part.

Careful consideration will be taken to maintain patient's confidentially. After consent is provided, an anonymous code will be assigned to each patient. Some patient details such as CHI number, age, gender and time of diagnosis, will need to be accessed by the research team, these details will be stored alongside patient code in a password-protected document.

At the end of the study, a lay summary of results will be provided to patients who have expressed a further interest. Project results will be made publically available on the Open Science Framework (https://osf.io/bxnqs/) within three months after study end-date (30/06/2020).

7 Footnotes

Author Contributions: Each author has contributed significantly one or more aspects of the study. All authors contributed to study development and design. RDM, SR and AGM were involved in implementation of study protocol and analysis design. All authors contributed to data acquisition for MCI and AD, with SP and MH leading patient recruitment. AGM and SR were involved in data acquisition for HC. AGM and RDM drafted the manuscript and all authors provided critical revisions and approved the final version.

Funding statement: This research is funded by the Dunhill Medical Trust Research Project Grant awarded to Prof Robert McIntosh (RPGF1810\86)

For peer review only

Competing interests: there are no conflicts or competing interests for AGM, SP, MH, SR or RDM.

8 References

1. Dubois B, Feldman HH, Jacova C, et al. Advancing research diagnostic criteria for Alzheimer's disease: the IWG-2 criteria. *Lancet Neurol*2014;13(6):614–29. doi.org/10.1016/S1474-4422(14)70090-0
2. Gordon BA, Blazey TM, Su Y et al. Spatial patterns of neuroimaging biomarker change in individuals from families with autosomal dominant Alzheimer's disease: a longitudinal study. *Lancet Neurol.*2018;17(3):211–2. doi.org/10.1016/S1474-4422(18)30028-0
3. Cavanna AE, Trimble MR. The precuneus: a review of its functional anatomy and behavioural correlates. *Brain*2006;129(3):564–83. doi.org/10.1093/brain/awl004
4. Karnath H-O, Perenin M-T. Cortical Control of Visually Guided Reaching: Evidence from Patients with Optic Ataxia. *Cereb Cortex*2005;15(10):1561–9. doi.org/10.1093/cercor/bhi034
5. Perenin MT, Vighetto A. Optic Ataxia: A Specific Disorder in Visuomotor Coordination. In: *Spatially Oriented Behavior*New York, NY: Springer New York; 1983 p. 305–26.
6. Rossetti Y, Pisella L, McIntosh RD. Definition: Optic ataxia. *Cortex*2019;in press.
7. Mathuranath PS, Nestor PJ, Berrios GE, et al. A brief cognitive test battery to differentiate Alzheimer's disease and frontotemporal dementia. *Neurology*2000;55(11):1613–20. doi.org/10.1212/01.wnl.0000434309.85312.19
8. Mathôt S, Schreij D, Theeuwes J. OpenSesame: An open-source, graphical experiment builder for the social sciences. *Behav Res Methods*2012;44(2):314–24. doi.org/10.3758/s13428-011-0168-7
9. Crawford JR, Howell DC. Comparing an Individual's Test Score Against Norms Derived from Small Samples. *Clin Neuropsychol*1998;12(4):482–6. doi.org/10.1076/clin.12.4.482.7241
10. Crawford JR, Garthwaite PH, Ryan K. Comparing a single case to a control sample:

For peer review only

Testing for neuropsychological deficits and dissociations in the presence of covariates. *Cortex*2011;47(10):1166–78. doi.org/10.1016/j.cortex.2011.02.017

11. Brainard DH. The Psychophysics Toolbox. *Spat Vis*1997;10:433–6. doi.org/10.1163/156856897X00357

9 Figure legends

Figure 1: 9 target positions during the lateral reaching task for left (A) and right (B) hand sides. At a viewing distance of 40cm targets are presented at approximately 28, 33 and 38° of eccentricity

Figure 2: target positions during the radial reaching task, shown here on both the right- and left-hand sides, at 11.4, 22.6, 33.4 and 43.6° from fixation. The start-button is positioned at the bottom of the screen 40cm away from central fixation. A webcam is placed at the point of central fixation (midpoint).

Figure 3: (a) Relation between control sample size and power to detect a single-case deficit in a one-tailed test, for different size of deficit (D, expressed as standard deviations of control mean), (b) Relation between deficit size (D) and power to detect a single case deficit, given a control sample size of 24, for adjusted and unadjusted alpha criteria.

For peer review only

A.

B.

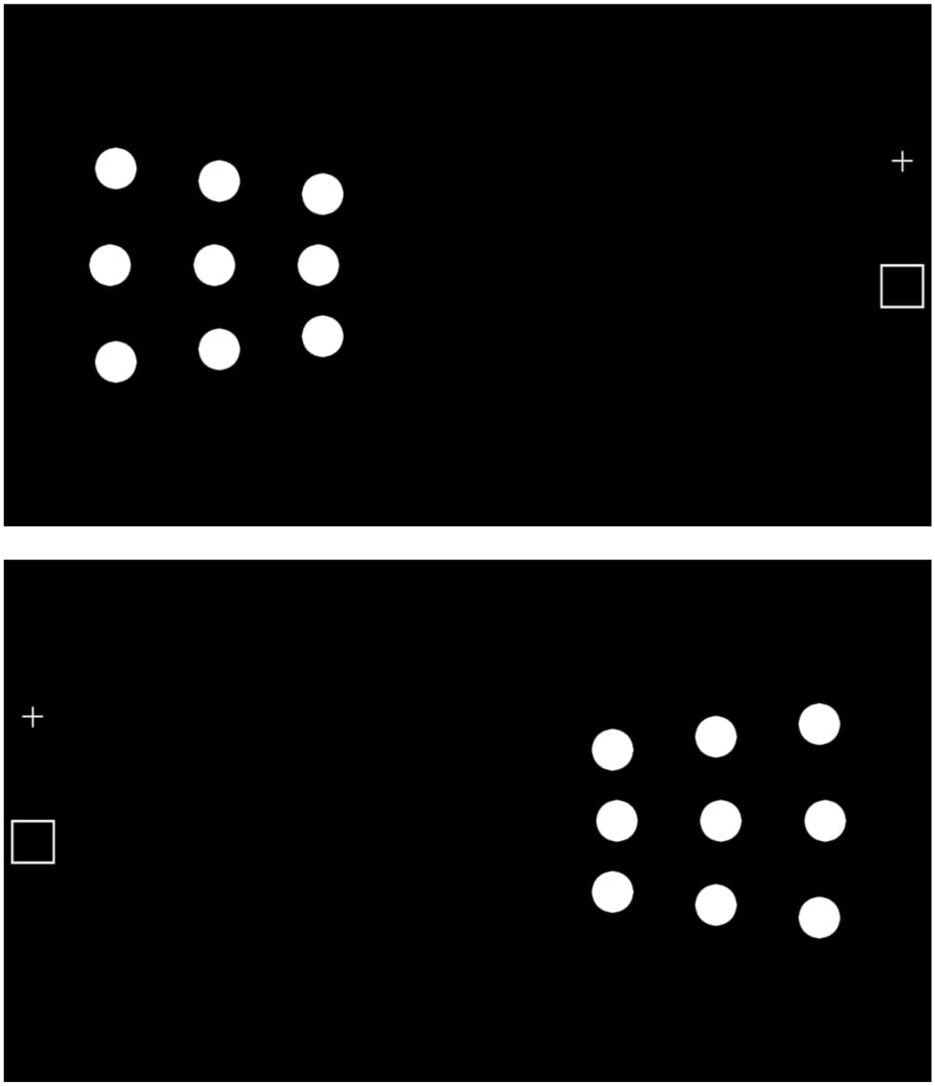

Figure 1: 9 target positions during the lateral reaching task for left (A) and right (B) hand sides. At a viewing distance of 40cm targets are presented at approximately 28, 33 and 38° of eccentricity

83x90mm (300 x 300 DPI)

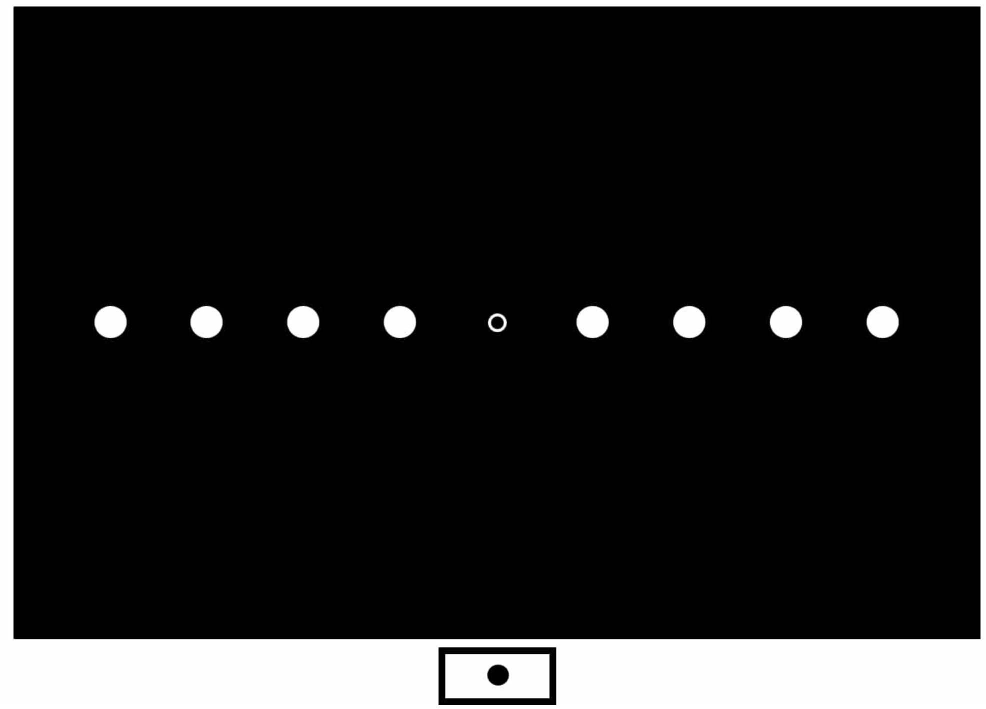

Figure 2: target positions during the radial reaching task, shown here on both the right- and left-hand sides, at 11.4, 22.6, 33.4 and 43.6° from fixation. The start-button is positioned at the bottom of the screen 40cm away from central fixation. A webcam is placed at the point of central fixation (midpoint).

112x80mm (300 x 300 DPI)

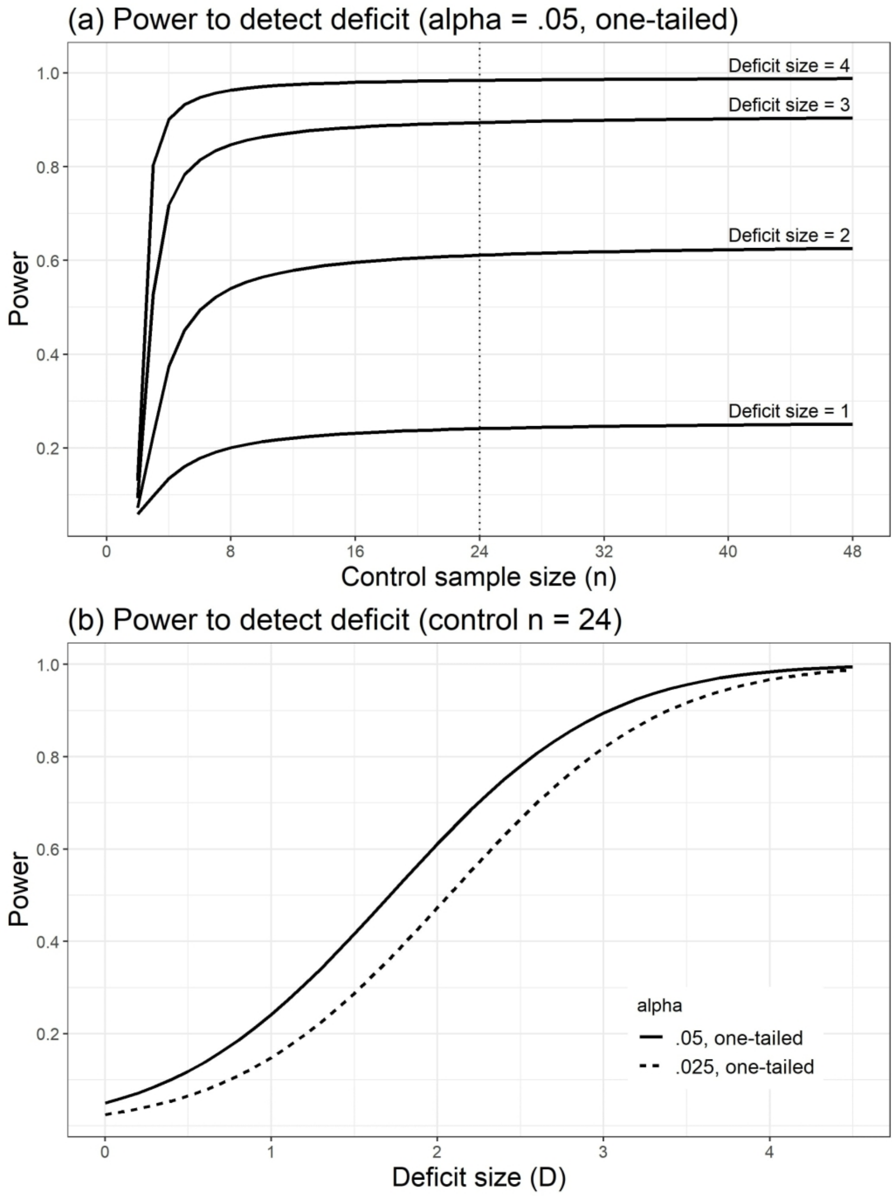

Figure 3: (a) Relation between control sample size and power to detect a single-case deficit in a one-tailed test, for different size of deficit (D, expressed as standard deviations of control mean), (b) Relation between deficit size (D) and power to detect a single case deficit, given a control sample size of 24, for adjusted and unadjusted alpha criteria.

190x254mm (300 x 300 DPI)

The assessment of visually guided misreaching in prodromal Alzheimer's disease: study protocol

Site-specific information

Site 1: Edinburgh

Patient recruitment in Edinburgh will take place at the Anne Rowling Regenerative Neurology Clinic (NHS Lothian), through a team led by Dr. Suvankar Pal. Patients who fit the recruitment criteria will be identified through the Rowling CARE-register and provided an information sheet and a notification of interest form.

All testing (patient and control) takes place in the Human Movement Laboratory, Department of Psychology, The University of Edinburgh.

Site 2: Norfolk

Patient recruitment will take place in the Julian Hospital in Norwich (NHS Norfolk & Suffolk). A team of research nurses will identify suitable participants who will be provided an information sheet and a notification of interest form.

All testing takes place in the Vision and Action Laboratory, Department of Psychology, The University of East Anglia.

For peer review only

STROBE Statement—checklist of items that should be included in reports of observational studies

	Item No	Recommendation
**Title and abstract**	1	(*a*) Indicate the study's design with a commonly used term in the title or the abstract
		(*b*) Provide in the abstract an informative and balanced summary of what was done and what was found
**Introduction**		
Background/rationale	2	Explain the scientific background and rationale for the investigation being reported
Objectives	3	State specific objectives, including any prespecified hypotheses
**Methods**		
Study design	4	Present key elements of study design early in the paper
Setting	5	Describe the setting, locations, and relevant dates, including periods of recruitment, exposure, follow-up, and data collection
Participants	6	(*a*) *Cohort study*—Give the eligibility criteria, and the sources and methods of selection of participants. Describe methods of follow-up<br>*Case-control study*—Give the eligibility criteria, and the sources and methods of case ascertainment and control selection. Give the rationale for the choice of cases and controls<br>*Cross-sectional study*—Give the eligibility criteria, and the sources and methods of selection of participants
		(*b*) *Cohort study*—For matched studies, give matching criteria and number of exposed and unexposed<br>*Case-control study*—For matched studies, give matching criteria and the number of controls per case
Variables	7	Clearly define all outcomes, exposures, predictors, potential confounders, and effect modifiers. Give diagnostic criteria, if applicable
Data sources/ measurement	8*	For each variable of interest, give sources of data and details of methods of assessment (measurement). Describe comparability of assessment methods if there is more than one group
Bias	9	Describe any efforts to address potential sources of bias
Study size	10	Explain how the study size was arrived at
Quantitative variables	11	Explain how quantitative variables were handled in the analyses. If applicable, describe which groupings were chosen and why
Statistical methods	12	(*a*) Describe all statistical methods, including those used to control for confounding
		(*b*) Describe any methods used to examine subgroups and interactions
		(*c*) Explain how missing data were addressed
		(*d*) *Cohort study*—If applicable, explain how loss to follow-up was addressed<br>*Case-control study*—If applicable, explain how matching of cases and controls was addressed<br>*Cross-sectional study*—If applicable, describe analytical methods taking account of sampling strategy
		(*e*) Describe any sensitivity analyses

Continued on next page

For peer review only - http://bmjopen.bmj.com/site/about/guidelines.xhtml

**Results**		
Participants	13*	(a) Report numbers of individuals at each stage of study—eg numbers potentially eligible, examined for eligibility, confirmed eligible, included in the study, completing follow-up, and analysed
		(b) Give reasons for non-participation at each stage
		(c) Consider use of a flow diagram
Descriptive data	14*	(a) Give characteristics of study participants (eg demographic, clinical, social) and information on exposures and potential confounders
		(b) Indicate number of participants with missing data for each variable of interest
		(c) *Cohort study*—Summarise follow-up time (eg, average and total amount)
Outcome data	15*	*Cohort study*—Report numbers of outcome events or summary measures over time
		Case-control study—Report numbers in each exposure category, or summary measures of exposure
		Cross-sectional study—Report numbers of outcome events or summary measures
Main results	16	(*a*) Give unadjusted estimates and, if applicable, confounder-adjusted estimates and their precision (eg, 95% confidence interval). Make clear which confounders were adjusted for and why they were included
		(*b*) Report category boundaries when continuous variables were categorized
		(*c*) If relevant, consider translating estimates of relative risk into absolute risk for a meaningful time period
Other analyses	17	Report other analyses done—eg analyses of subgroups and interactions, and sensitivity analyses
**Discussion**		
Key results	18	Summarise key results with reference to study objectives
Limitations	19	Discuss limitations of the study, taking into account sources of potential bias or imprecision. Discuss both direction and magnitude of any potential bias
Interpretation	20	Give a cautious overall interpretation of results considering objectives, limitations, multiplicity of analyses, results from similar studies, and other relevant evidence
Generalisability	21	Discuss the generalisability (external validity) of the study results
**Other information**		
Funding	22	Give the source of funding and the role of the funders for the present study and, if applicable, for the original study on which the present article is based

*Give information separately for cases and controls in case-control studies and, if applicable, for exposed and unexposed groups in cohort and cross-sectional studies.

Note: An Explanation and Elaboration article discusses each checklist item and gives methodological background and published examples of transparent reporting. The STROBE checklist is best used in conjunction with this article (freely available on the Web sites of PLoS Medicine at http://www.plosmedicine.org/, Annals of Internal Medicine at http://www.annals.org/, and Epidemiology at http://www.epidem.com/). Information on the STROBE Initiative is available at www.strobe-statement.org.

For peer review only

BMJ Open

The assessment of visually guided reaching in prodromal Alzheimer's disease: a cross-sectional study protocol

Journal:	*BMJ Open*
Manuscript ID	bmjopen-2019-035021.R1
Article Type:	Protocol
Date Submitted by the Author:	26-Feb-2020
Complete List of Authors:	Mitchell, Alexandra; The University of Edinburgh, School of Psychology, Philosophy & Language Sciences<br>McIntosh, Robert; The University of Edinburgh, School of Psychology, Philosophy & Language Sciences<br>Rossit, Stephanie; University of East Anglia, School of Psychology<br>Hornberger, M; University of East Anglia, School of Medicine<br>Pal, Suvankar; The University of Edinburgh, Anne Rowling Regenerative Neurology Clinic, Centre for Clinical Brain Sciences
**Primary Subject Heading**:	Neurology
Secondary Subject Heading:	Mental health, Pathology
Keywords:	Dementia < NEUROLOGY, Adult neurology < NEUROLOGY, Neurophysiology < NEUROLOGY, NEUROPATHOLOGY

SCHOLARONE™
Manuscripts

I, the Submitting Author has the right to grant and does grant on behalf of all authors of the Work (as defined in the below author licence), an exclusive licence and/or a non-exclusive licence for contributions from authors who are: i) UK Crown employees; ii) where BMJ has agreed a CC-BY licence shall apply, and/or iii) in accordance with the terms applicable for US Federal Government officers or employees acting as part of their official duties; on a worldwide, perpetual, irrevocable, royalty-free basis to BMJ Publishing Group Ltd ("BMJ") its licensees and where the relevant Journal is co-owned by BMJ to the co-owners of the Journal, to publish the Work in this journal and any other BMJ products and to exploit all rights, as set out in our licence.

The Submitting Author accepts and understands that any supply made under these terms is made by BMJ to the Submitting Author unless you are acting as an employee on behalf of your employer or a postgraduate student of an affiliated institution which is paying any applicable article publishing charge ("APC") for Open Access articles. Where the Submitting Author wishes to make the Work available on an Open Access basis (and intends to pay the relevant APC), the terms of reuse of such Open Access shall be governed by a Creative Commons licence – details of these licences and which Creative Commons licence will apply to this Work are set out in our licence referred to above.

Other than as permitted in any relevant BMJ Author's Self Archiving Policies, I confirm this Work has not been accepted for publication elsewhere, is not being considered for publication elsewhere and does not duplicate material already published. I confirm all authors consent to publication of this Work and authorise the granting of this licence.

For peer review only

The assessment of visually guided reaching in prodromal Alzheimer's disease: a cross-sectional study protocol

Alexandra G. Mitchell[1], *Robert D. McIntosh*[1], *Stephanie Rossit*[2], *Michael Hornberger*[3,4] & *Suvankar Pal*[5]

[1] School of Psychology, Philosophy & Language Sciences, University Of Edinburgh, Edinburgh, UK

[2] School of Psychology, University of East Anglia, Norwich UK

[3] Department of Medicine, Norwich Medical School, Univeristy of East Anglia, Norwich, UK

[4] Norfolk and Suffolk NHS Foundation Trust, Norwich, UK

[5] Anne Rowling Regenerative Neurology Clinic, Centre for Clinical Brain Sciences, University of Edinburgh, Edinburgh, UK

Corresponding author:

Dr Alexandra G. Mitchell

7 George Square,

Edinburgh,

EH8 9JZ,

UK

alexandra.mitchell@ed.ac.uk

+44(0)131 651 3232

Word count: 3,593

For peer review only

For peer review only - http://bmjopen.bmj.com/site/about/guidelines.xhtml

1 Abstract

Introduction: Recent evidence has implicated the precuneus of the medial parietal lobe as one of the first brain areas to show pathological changes in Alzheimer's disease (AD). Damage to the precuneus through focal brain injury is associated with impaired visually guided reaching, particularly for objects in peripheral vision. This raises the hypothesis that peripheral misreaching may be detectable in patients with prodromal AD. The aim of this study is to assess the frequency and severity of peripheral misreaching in patients with mild cognitive impairment (MCI) and AD.

Methods and analysis: Patients presenting with amnestic MCI, mild-to-moderate AD, and healthy older-adult controls will be tested (target N=24 per group). Peripheral misreaching will be assessed using two set ups: a tablet-based task of lateral reaching, and motion-tracked radial reaching (in depth). There are two versions of each task, where participants can look directly at targets (free reaching), and when they must maintain central fixation (peripheral reaching). All tasks will be conducted first on their dominant and then their non-dominant side. For each combination of task and side, a peripheral misreaching index (PMI) is then calculated as the increase in absolute reaching error between free and peripheral reaching. Each patient will be classified as showing peripheral misreaching if their PMI is significantly abnormal, by comparison to control performance on either side of space. We will then test whether the frequency of peripheral misreaching exceeds the chance level in each patient group and compare the overall severity of misreaching between groups.

Ethics and dissemination: Ethical approval was provided by the NHS East of England, Cambridge Central Research Ethics Committee (REC 19/EE/0170). The results of this study will be published in a peer reviewed journal and presented at academic conferences.

Key words: Alzheimer's disease, cognitive impairment, visually guided action, peripheral reaching, optic ataxia

For peer review only

2 Article Summary

Strengths and Limitations of this study

- The first study to systematically assess visually guided reaching in patients with cognitive impairment
- Includes a simple tablet-based task (lateral reaching) that could be readily translated to clinical settings to assess the presence of peripheral misreaching
- Case-control statistical tests of deficit are inherently low-powered, subtle deficits of misreaching may not be detected at the level of individual patients

For peer review only

For peer review only - http://bmjopen.bmj.com/site/about/guidelines.xhtml

3 Introduction

The pathophysiological cascade that leads to Alzheimer's disease (AD) can begin up to 20 years before the onset of cognitive problems in both autosomal and sporadic AD (1–5). In dominant and early onset cases, there is evidence that the precuneus is one of the earliest regions to be affected (6,7). Focal damage in and around this brain area is known to be associated with deficits of visually guided action (8). One example of such a condition is optic ataxia, an impairment of misreaching typically reflected in peripheral vision (9,10). Patients with optic ataxia often do not often complain of this symptom and it is rarely assessed in clinical settings, and it can therefore go undetected (11). The changes observed in the precuneus in prodromal AD, and the link between the precuneus and optic ataxia, raise the hypothesis that optic ataxic misreaching may be detectable in patients with prodromal AD.

3.1 Specific hypothesis

The hypothesis that peripheral misreaching is a feature of AD predicts that individual patients with AD, and possibly those with MCI, will show an abnormally large inflation of reaching errors when aiming for targets in peripheral vision, as compared with targets in free vision. At a group level, patients with AD and, to a lesser extent, patients with MCI will show significantly greater peripheral misreaching than healthy controls.

For peer review only

4 Methods

4.1 Study setting

The study is a collaboration between clinicians and University staff at the University of Edinburgh (UoE) and University of East Anglia (UEA). Details of recruitment and site information can be found in the supplementary materials. Data collection for this study began on 03/10/2019 and 8/48 patients have taken part. Data for healthy controls have already been collected.

4.2 Participants

Patients with a diagnosis of amnestic MCI or typical (amnestic) mild-to-moderate AD will be invited to take part. Mild to moderate AD will be defined by a score of at least 50 in the most recent administration of Addenbrooke's Cognitive Examination (ACE-III) (12) If there is no recorded ACE-III score, clinical opinion of patient's condition will be used to assess eligibility.

Older adults without any known neurological disorders will be tested as a healthy control (HC) group. To achieve our target of 24 full data-sets per group (*Section 5.3*), we plan to test up to 30 participants in each group, allowing for possible withdrawals.

4.2.1 Inclusion criteria

For all participant groups, the ability to give informed consent is the initial inclusion criterion. Additional inclusion criteria are then applied to each group.

Control group inclusion criteria:

- Aged 50 – 75[1]
- No reported neurological or neurodegenerative conditions

MCI group inclusion criteria:

- Aged 45 – 85

[1] NB. The age-range for controls is targeted at the expected age range for patients, but the allowable range of ages for patients is wider than this, in order not to restrict recruitment unnecessarily.

For peer review only - http://bmjopen.bmj.com/site/about/guidelines.xhtml

- Clinical diagnosis of MCI with an amnestic pattern of presentation. This includes an observed deficit on cognitive/neuropsychological testing suggesting amnestic and visuospatial profile deficit, low β-amyloid, elevated phosphorylated Tau, regional atrophy on MR brain imaging and/or regional perfusion changes on HMPAO-SPECT

AD group inclusion criteria:

- Aged 45 – 85
- Clinical diagnosis of AD

4.2.2 Exclusion criteria

For all participant groups, the following exclusion criteria are applied:

- Significant difficulty communicating or understanding instructions in English
- Significant, uncorrected visual impairment (e.g., cataract, macular degeneration, scotoma, amblyopia, strabismus)
- Conditions that could interfere with smooth hand movements (e.g. ataxia, essential tremor, severe arthritis)
- Prior history of stroke or TIA
- Clinical features suggested of Lewy body pathology (e.g. visual hallucinations or REM sleep disorder)

4.2.3 Public and Patient involvement

Patients with MCI or AD and their careers were involved in the early stages of planning and development. A focus group was held at the Anne Rowling Clinic in Edinburgh where patients and carers had the opportunity to try out prototypes of the tablet-based reaching task and provide feedback on task design. This feedback was used to optimise the final task for patient accessibility and clarity.

4.3 Tasks

Two different set-ups will be used to assess peripheral reaching: a tablet-based assessment of reaching in the frontoparallel plane (lateral reaching), and a motion-tracking assessment of reaching in radial depth (radial reaching). Participants will complete two versions of each reaching task: a version in which participants look

For peer review only - http://bmjopen.bmj.com/site/about/guidelines.xhtml

directly at targets before reaching to them (free reaching); and a version where central fixation is maintained (peripheral reaching). Any general factors affecting motor accuracy should influence both free and peripheral reaching, so we will treat the free reaching condition as a baseline condition, to be subtracted from peripheral reaching performance, to isolate the specific increase in error due to peripheral target presentation (13). The critical outcome measure is therefore the inflation of absolute reaching error in peripheral reaching relative to free reaching.

Before testing, the participant's dominant writing hand is identified (by self-report). All tasks are completed first on the dominant side, using the dominant hand, followed by the non-dominant side and hand. Lateral reaching is completed first, followed by radial reaching. All tasks are performed in the same order for all participants.

4.3.1 Lateral reaching tasks

i. Stimuli & Apparatus

Stimuli are presented on a HP Pavillion x360 touch screen (active display 310x175mm, resolution 1920x1080 pixels). Tasks are controlled by a custom program written in OpenSesame version 3.2.8 *Kafkesque Koffka* (14). Participants are seated 40cm away from the screen which is positioned with either the right edge of the screen aligned to their midline (left-sided reaching, Figure 1A) or the left edge (right-sided reaching, Figure 1B). A start box (white rectangle, 2x2°, 13.96x13.96mm) is drawn at the centre edge (right or left) of the screen, aligned to participant's midpoint. In some tasks (detailed below) a white fixation cross is present (1x1°, 6.98x6.98mm), located 34.9mm (5°) directly above the start box. Targets are white circles (diameter = 2°, 13.96mm) presented along radial spokes at 28, 33 and 38° to the left (Figure 1A) or right (Figure 1B) of fixation. The experimenter sits across the table and monitors eye movements directly.

ii. Free reaching

For the first block in the lateral reaching task participants are not required to fixate, therefore the fixation cross is absent.

For peer review only

Participants initiate a trial by pressing and holding down the start box, which disappears at touch. At this point they are may search the screen for a target. After a short delay (250-750ms, randomised 100ms intervals) a target appears at one of nine possible locations. As soon as the target appears, participants look at it and make one smooth reach to try to touch the target. The target remains on the screen until a touch is recorded at any location, then the target disappears and a short beep (100ms, 440Hz) is played. The validity of the trial is then coded by the experimenter using a keyboard; 'y' – valid trial, 'e' – the participant did not move their eyes to the target, 'v' – invalid trial, and the start box reappears to begin another trial.

If the experimenter presses 'e' or 'v' that trial is repeated until a valid trial is recorded. The block ends after a minimum of 27 valid trials (3 per target location), or after 50 valid and 'no eye-movement' trials.

iii. Visual detection

This is a simple check to confirm that the participant is capable of detecting the targets when presented in peripheral vision, to be allow for a meaningful test of peripheral reaching (*Section 4.3.1iv*).

Throughout each trial the participant must gaze at the fixation cross. They initiate the trial by pressing and holding down the start box, which disappears when touched. In order to aid the maintenance of fixation, the fixation cross cycles between white and red at the screen refresh rate (60Hz). After a short delay (250-750ms), a target can appear at one of the nine locations for one second, or no target appears. This is followed by a short beep (100ms, 440Hz) to indicate the end of the trial. The participant must verbally report whether or not a target was seen in that interval. The experimenter records the response using the keyboard ('y' – yes, 'n' – no). If the participant makes an eye-movement, the experimenter presses 'e' and the trial is repeated. The block ends after 15 valid (no eye-movement) trials, one for each of the nine target locations, and six catch trials with no target.

For peer review only

For peer review only - http://bmjopen.bmj.com/site/about/guidelines.xhtml

To progress to the peripheral reaching task, participants are required to detect at least 6/9 targets and correctly rejects at least 3/6 catch trials. Otherwise, testing is discontinued on that side of space.

iv. Peripheral reaching

For peripheral reaching participants are required to gaze at the fixation cross throughout each trial. A trial begins by pressing and holding down the start box. When touched, the start box disappears and the fixation cross cycles between white and red (at a rate of 60Hz) until the trial ends. After a short delay (250-750ms) a target appears at one of nine locations. Whilst maintaining fixation, participants make one smooth reaching movement to try to touch the target. The target remains on the screen until a touch is recorded at any location, and a short beep is played once the target disappears. The experimenter then records the validity of the trial; 'y' – valid, 'e' – participant moved their eyes away from fixation, 'v' – invalid trial.

Invalid ('e' or 'v') trials are repeated until a valid trial is recorded. The block ends after a minimum of 27 valid trials (3 per target location), or after 50 valid and 'eye-movement' trials.

4.3.2 Radial reaching tasks

i. *Stimulus & Apparatus*[2]

An infrared motion-tracking camera (Optotrak Certus, Northern Digital Inc) is used to track the reaching movement. Infra-red-emitting diodes (IREDs) are taped to the tip of the right and left index fingers of each participant to track the reach in each hand. The Optotrak samples the IRED's 3D position at 100Hz throughout each 2000ms trial. The task is controlled by custom software written in LabView (National Instruments) programming environment.

---

[2] The stimuli and apparatus reported here are specific to UoE. At the second site, UEA, motion tracking was performed a Qualisys AB system (Gothenburg, Sweden). Targets are green LEDs (diameter = 0.68°, 6mm). The experiment is presented on a grey table (100x100cm) and the experiment is run through Psychophysics Toolbox (15) in MATLAB (Mathworks, USA)

For peer review only - http://bmjopen.bmj.com/site/about/guidelines.xhtml

Participants are seated with their head placed in a chin-rest in line with the middle of the display. Stimuli are back-projected via a mirror onto a screen (1000mm wide x 750mm deep) that lies flat in-front of the participant. A webcam is placed on the screen 50cm directly in-front of the participant, as a fixation point. The live webcam image feeds into a separate laptop, allowing experimenter to monitor gaze. A start-button is aligned to the centre of the screen, positioned 10cm in-front of the participant, 40cm away from fixation (Figure 2). Targets are white circles (diameter = 1.60°, 13.96mm) presented at 4 eccentric locations (11.4, 22.6, 33.4 and 43.6° away from centre) on each side (Figure 2).

ii. Calibration

A calibration procedure is carried out before the reaching tasks to record the IRED position at the actual target location. A target is displayed at one target location and the participant is instructed to cover it completely with their reaching finger. Once the target is covered, the experimenter presses the start button and the finger location is recorded for 2000ms. A beep plays after 2000ms, indicating that the participant can move their hand away from the target position. Another target appears at the next location and the same procedure is repeated. Calibration is run using the ipsilateral hand for four targets on the left side, and four on the right.

iii. Free reaching

Participants initiate a trial by pressing and holding down the start button. As soon as they push the button down, participants may look around the screen for a target. After 250-750ms a target appears, participants then look directly at the target and reach to touch the target in one smooth movement. Optotrak recording is initiated simultaneously with target appearance, and the target disappearance is the simultaneous with the end of the recording after 2000ms. When the target disappears a short beep (100ms, 440Hz) plays, the participant leaves their finger at its landing position until they hear the beep. After the trial, the experimenter codes the trial validity with a key-press; 'Return' – valid, 'F1' – no eye-movement, 'Esc' – invalid trial. If

For peer review only

an invalid trial (‘F1’ or ‘Esc’) is coded the trial gets recycled to the end of the shuffled trial.

The block ends once 28 valid trials (7 per target location) are recorded, or after 50 valid and ‘no eye-movement’ trials.

iv. Peripheral reaching

To assess reaching accuracy in the periphery participants are required to look directly at central fixation (the webcam) throughout each trial. Participants initiate a trial by pressing and holding down the start button. After 250-750ms a target appears. Whilst maintaining gaze on the webcam participants make one smooth reaching movement to try to touch the target. After the reach, participants leave their finger at its landing position until a short beep (100ms, 440Hz). The target remains on screen for 2000ms after the trial begins. The motion-tracker records the reach throughout the 2000ms trial. At the end of the trial, the experimenter codes trial validity; ‘Return’ – valid trial, ‘F1’ – eye movement during trial, ‘Esc’ – invalid trial. If an invalid trial (‘F1, ‘Esc’) is recorded then the trial is recycled to the end of the shuffled trial list.

The block ends after 28 valid trials (7 per target location) are recorded, or after 50 valid and ‘eye-movement’ trials.

For peer review only - http://bmjopen.bmj.com/site/about/guidelines.xhtml

5 Analysis plan

5.1 Lateral reaching task

For the critical analyses, a single measure of *reaching accuracy* is taken for each participant, for each combination of viewing condition (free, peripheral) and side (non-dominant, dominant). For each response, the absolute error (in mm, x and y axis) is recorded as the distance of the reach endpoint from the target midpoint. The median absolute error is then calculated for each target eccentricity, across responses to the 3 targets at that eccentricity, for each combination of viewing condition and side. The average absolute error is then calculated as the mean of the medians for the 3 eccentricities, to give the single measure of reaching accuracy for each viewing condition at each side.

For the comparison of individual patients against control performance, the data are further compressed to a single index of performance per side, by subtracting reaching accuracy in the free vision condition from the peripheral condition. We call this value the *peripheral misreaching index (PMI)*.

5.1.1 Analysis of single case deficits

We will screen the control group for outliers that might suggest abnormalities, as such values would reduce the (already low, see Figure 3) power to detect single-case deficits. We will use a robust method of outlier detection based on the median absolute deviation (MAD). The MAD can be multiplied by the consistency constant 1.4826 to estimate the standard deviation, assuming a normal distribution. Each control participant's PMI can be expressed a modified Z-score (Z') by subtracting the group median, divided by the median absolute deviation *1.4826. If Z' exceeds 2.5, that participant will be excluded, and replaced. Our simulations suggest that, for a group size of 24, we would expect to exclude (on average) < 1 participant (~0.67) by this criterion.

We will next assess, for each side, whether the PMI of controls is related to age or sex, by computing Pearson's correlations. If the correlation is ≥ .3 on either side, then that variable will be included as a covariate in the subsequent case-control comparisons for both sides.

For peer review only

For peer review only - http://bmjopen.bmj.com/site/about/guidelines.xhtml

Case-control comparisons will then be run to compare each patient's PMI against control performance. These comparisons will be based upon Crawford & Howell's (16) modified t-test; or, if covariates are included, we will use the updated test of deficit (17). The individual tests will be one-tailed, with an alpha-level set to .025, in order to constrain per-patient alpha level (across the two sides) to .05. If a patient shows a deficit on either side that would meet the unadjusted criterion (.05), but not the adjusted criterion (.025), they will be classified as showing borderline peripheral misreaching.

Finally, a binomial test will test whether the rate of observed peripheral misreaching exceeds the rate expected by chance (i.e. the per-patient adjusted alpha level of .05). A significant outcome ($p < .05$) for either patient group will indicate that peripheral misreaching is a feature of this patient group. The observed rate of peripheral misreaching will provide an estimate of how common it is. We will run a further analysis including borderline cases and compare the rate of peripheral misreaching in each patient group against the appropriate chance level of .10.

5.1.2 Group-level analysis

The case control approach will be complemented by a group-level ANOVA of reaching accuracy, as measured by the PMI, with the between-subject factor of group (HC, MCI, AD) and the within subject factor of side (non-dominant, dominant). This analysis will test whether the average severity of peripheral misreaching in each patient group significantly exceeds that observed in healthy controls.

5.1.3 Exploratory analyses

Any lateralisation that occurs in MCI/AD is likely to be limited, therefore, any impairment in peripheral reaching may be similarly non-lateralised. An average PMI (across both sides) will therefore be calculated to assess peripheral reaching ability overall. More detailed analyses will be run with a between subject factor of group and within subject factors of side, eccentricity and viewing condition. These analyses will be conducted using dependent variables of absolute reaching error, directional (signed) reaching error, reaction time and movement time. The expectation is that peripheral

For peer review only - http://bmjopen.bmj.com/site/about/guidelines.xhtml

misreaching will manifest as a fixation-directed bias, which is exacerbated at higher eccentricities significantly more so in patient groups than in age-matched controls.

5.2 Radial reaching task

IRED speed is used to determine onset and offset of the reaching movement. Movement onset is defined as the first frame in which the IRED's speed exceeds 50mm/s (and maintains that speed for up to 100ms). Movement offset is defined as the first subsequent frame in which IRED speed falls below 50mm/s. The landing position of the movement is defined by the x and y coordinates in the final frame of the movement and will be recorded as errors relative to true target locations recorded during calibration for each participant.

An initial analysis of PMI for the radial reaching task will be performed, restricted to the two most eccentric target positions (33.4 and 43.6°). Case-control comparisons follow the plan for the lateral reaching task (*Section 5.1.1*), to estimate the rates of peripheral misreaching, and borderline peripheral misreaching, in the two patient groups. Due to different experimental set ups between the two test sites (UoE, UEA), each patient will be referenced to the same-site control data for case-control comparisons.

A group level ANOVA of PMI, restricted to the two most eccentric target positions, will similarly follow the plan for lateral reaching (*Section 5.1.2*). We will include site (UoE, UEA) as an additional covariate. Subsequent, more detailed analyses will also follow the plan for lateral reaching (*Section 5.1.2*). Since motion tracking also provides kinematic variables on reaching trajectories, we also aim to examine the dependent variables peak speed and time to peak speed, normalised time after peak speed until reach endpoint and number of secondary movements.

5.3 Power considerations

The target sample sizes (N=24 per group) are based on power considerations related to the main inferential analyses, the case-control comparisons, and binomial tests of rates of peripheral misreaching deficits for the lateral reaching task.

For peer review only

The control sample size of 24 will provide close to the maximum power for case-control tests of deficit (figure 3A). Note that high power for these comparisons is inherently unachievable unless the deficit being tested for is very large. We do not know how large any misreaching deficits may be in our patient groups, but our control sample size will provide close to the maximum achievable power to detect them if they exist. Figure 3B illustrates more fully the relationship between deficit size (D) and power, for the adjusted alpha level (.025) and unadjusted alpha level (.05) by which we will determine peripheral misreaching deficits and borderline cases respectively (*Section 5.1.1*).

The main hypothesis is that peripheral misreaching will be found in a significant proportion of patients with AD and MCI. For one-sample binomial test to determine whether the observed rate of peripheral misreaching exceeds the chance level of .05, a sample size of 24 has > .9 power. Provided that the true population proportion is at least .2 (1 in 5). This is appropriate to our aims, since peripheral misreaching would be of limited significance in these clinical populations if its prevalence were less than 1 in 5.

For peer review only

For peer review only - http://bmjopen.bmj.com/site/about/guidelines.xhtml

6 Ethics and dissemination

This protocol was approved by UK Health Research Authority, by the East of England, Cambridge Central Research Ethics Committee on 13 June 2019 (REC reference 19/EE/0170).

All patients will provide informed consent, highlighting the voluntary nature of the study and their right to withdraw. If there is any doubt about the ability of the patient to provide informed consent, then this patient will not be recruited. There are no direct risks associated with taking part.

Careful consideration will be taken to maintain patient's confidentially. After consent is provided, an anonymous code will be assigned to each patient. Some patient details such as CHI number, age, gender and time of diagnosis, will need to be accessed by the research team, these details will be stored alongside patient code in a password-protected document.

At the end of the study, a lay summary of results will be provided to patients who have expressed a further interest. Project results will be made publicly available on the Open Science Framework (https://osf.io/bxnqs/) within three months after study end date (30/06/2020). Alongside this, we plan to publish the results of this protocol will be published in a peer reviewed journal and presented at academic conferences.

7 Footnotes

Author Contributions: Each author has contributed significantly one or more aspects of the study. All authors contributed to study development and design. RDM, SR and AGM were involved in implementation of study protocol and analysis design. All authors contributed to data acquisition for MCI and AD, with SP and MH leading patient recruitment. AGM and SR were involved in data acquisition for HC. AGM and RDM drafted the manuscript and all authors provided critical revisions and approved the final version.

For peer review only

Funding statement: This research is funded by the Dunhill Medical Trust Research Project Grant awarded to Prof Robert McIntosh (RPGF1810\86)

Competing interests: there are no conflicts or competing interests for AGM, SP, MH, SR or RDM.

8 References

1. Dubois B, Feldman HH, Jacova C, Hampel H, Molinuevo JL, Blennow K, et al. Advancing research diagnostic criteria for Alzheimer's disease: the IWG-2 criteria. Lancet Neurol [Internet]. 2014 Jun 1 [cited 2019 Aug 29];13(6):614–29. Available from: https://www.sciencedirect.com/science/article/pii/S1474442214700900
2. Pike KE, Savage G, Villemagne V, Ng S, Moss S, Maruff P, et al. β-amyloid imaging and memory in non-demented individuals: evidence for preclinical Alzheimer's disease. Brain [Internet]. 2007 [cited 2020 Feb 20];130(11):2837–44. Available from: https://academic.oup.com/brain/article/130/11/2837/331929
3. Jack CR, Knopman DS, Jagust WJ, Petersen RC, Weiner MW, Aisen PS, et al. Tracking pathophysiological processes in Alzheimer's disease: An updated hypothetical model of dynamic biomarkers. Vol. 12, The Lancet Neurology. Elsevier; 2013. p. 207–16.
4. Villemagne VL, Burnham S, Bourgeat P, Brown B, Ellis KA, Salvado O, et al. Amyloid β deposition, neurodegeneration, and cognitive decline in sporadic Alzheimer's disease: A prospective cohort study. Lancet Neurol. 2013 Apr 1;12(4):357–67.
5. Gordon BA, Blazey TM, Su Y, Hari-Raj A, Dincer A, Flores S, et al. Spatial patterns of neuroimaging biomarker change in individuals from families with autosomal dominant Alzheimer's disease: a longitudinal study. Lancet Neurol [Internet]. 2018 Mar 1 [cited 2019 Mar 27];17(3):241–50. Available from: https://www.sciencedirect.com/science/article/pii/S1474442218300280
6. Gordon BA, Blazey TM, Su Y, Hari-Raj A, Dincer A, Flores S, et al. Spatial patterns of neuroimaging biomarker change in individuals from families with autosomal dominant Alzheimer's disease: a longitudinal study. Lancet Neurol. 2018;17(3):211–2.
7. Möller C, Vrenken H, Jiskoot L, Versteeg A, Barkhof F, Scheltens P, et al. Different

For peer review only

patterns of gray matter atrophy in early- and late-onset Alzheimer's disease. Neurobiol Aging. 2013 Aug 1;34(8):2014–22.

8. Cavanna AE, Trimble MR. The precuneus: a review of its functional anatomy and behavioural correlates. Brain [Internet]. 2006 Mar 1 [cited 2019 Aug 29];129(3):564–83. Available from: http://academic.oup.com/brain/article/129/3/564/390904/The-precuneus-a-review-of-its-functional-anatomy

9. Karnath H-O, Perenin M-T. Cortical Control of Visually Guided Reaching: Evidence from Patients with Optic Ataxia. Cereb Cortex [Internet]. 2005 Oct 1 [cited 2019 Aug 29];15(10):1561–9. Available from: http://academic.oup.com/cercor/article/15/10/1561/396841/Cortical-Control-of-Visually-Guided-Reaching

10. Perenin MT, Vighetto A. Optic Ataxia: A Specific Disorder in Visuomotor Coordination. In: Spatially Oriented Behavior [Internet]. New York, NY: Springer New York; 1983 [cited 2019 Aug 29]. p. 305–26. Available from: http://link.springer.com/10.1007/978-1-4612-5488-1_17

11. Rossetti Y, Pisella L, McIntosh RD. Definition: Optic ataxia. Cortex. 2019;in press.

12. Mathuranath PS, Nestor PJ, Berrios GE, Rakowicz W, Hodges JR. A brief cognitive test battery to differentiate Alzheimer's disease and frontotemporal dementia. Neurology [Internet]. 2000 Dec 12 [cited 2019 Oct 15];55(11):1613–20. Available from: http://www.ncbi.nlm.nih.gov/pubmed/8190290

13. Borchers S, Müller L, Synofzik M, Himmelbach M. Guidelines and quality measures for the diagnosis of optic ataxia. Front Hum Neurosci [Internet]. 2013 Jul 2 [cited 2019 Mar 27];7:324. Available from: http://journal.frontiersin.org/article/10.3389/fnhum.2013.00324/abstract

14. Mathôt S, Schreij D, Theeuwes J. OpenSesame: An open-source, graphical experiment builder for the social sciences. Behav Res Methods [Internet]. 2012 Jun 16 [cited 2019 Oct 10];44(2):314–24. Available from: http://www.springerlink.com/index/10.3758/s13428-011-0168-7

15. Brainard DH. The Psychophysics Toolbox. Spat Vis [Internet]. 1997 [cited 2019 Oct 11];10:433–6. Available from: http://color.psych.upenn.edu/brainard/PsychToolbox.pdf

16. Crawford JR, Howell DC. Comparing an Individual's Test Score Against Norms

For peer review only

For peer review only - http://bmjopen.bmj.com/site/about/guidelines.xhtml

Derived from Small Samples. Clin Neuropsychol [Internet]. 1998 Nov 9 [cited 2019 Sep 13];12(4):482–6. Available from: https://www.tandfonline.com/doi/full/10.1076/clin.12.4.482.7241

17. Crawford JR, Garthwaite PH, Ryan K. Comparing a single case to a control sample: Testing for neuropsychological deficits and dissociations in the presence of covariates. Cortex. 2011;47(10):1166–78.

9 Figure legends

Figure 1: 9 target positions during the lateral reaching task for left (A) and right (B) hand sides. At a viewing distance of 40cm targets are presented at approximately 28, 33 and 38° of eccentricity

Figure 2: target positions during the radial reaching task, shown here on both the right- and left-hand sides, at 11.4, 22.6, 33.4 and 43.6° from fixation. The start-button is positioned at the bottom of the screen 40cm away from central fixation. A webcam is placed at the point of central fixation (midpoint).

Figure 3: (a) Relation between control sample size and power to detect a single-case deficit in a one-tailed test, for different size of deficit (D, expressed as standard deviations of control mean), (b) Relation between deficit size (D) and power to detect a single case deficit, given a control sample size of 24, for adjusted and unadjusted alpha criteria.

For peer review only

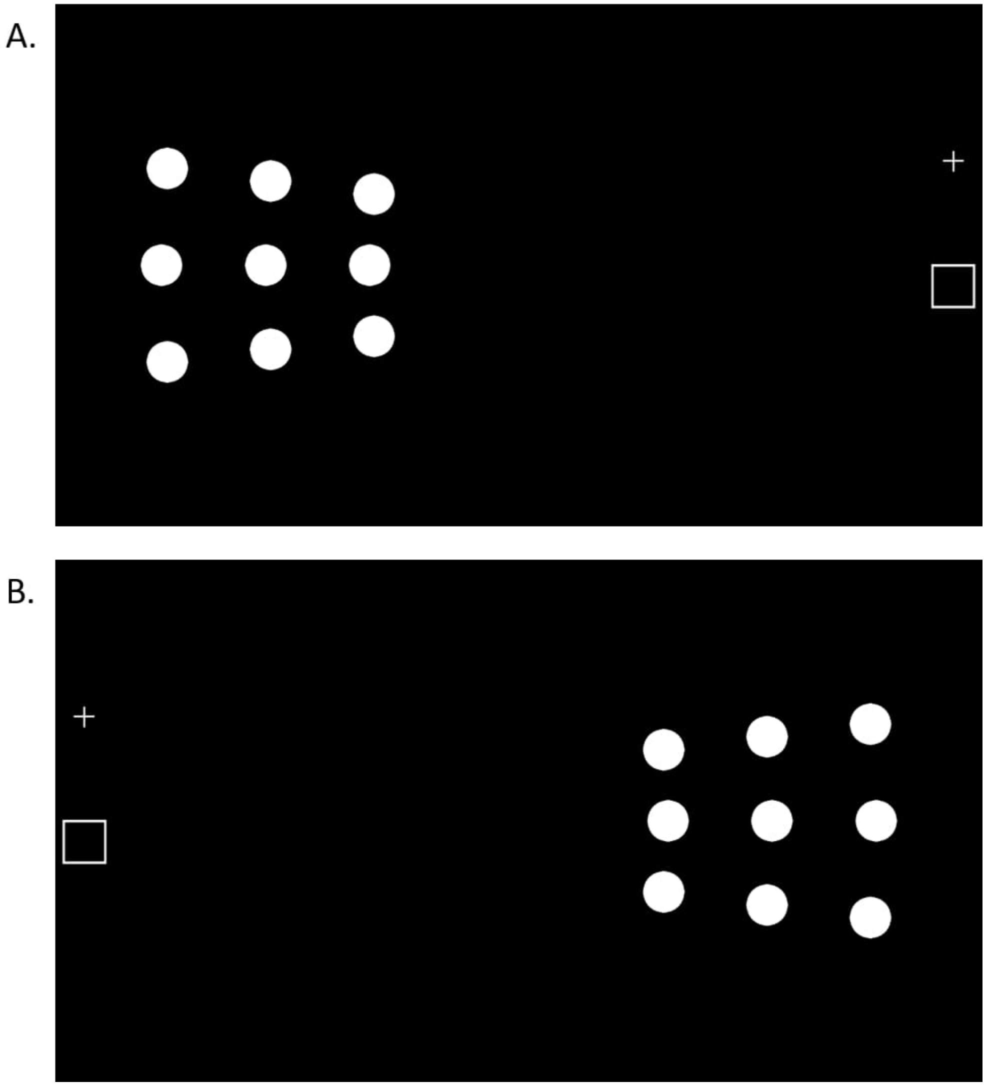

Figure 1: 9 target positions during the lateral reaching task for left (A) and right (B) hand sides. At a viewing distance of 40cm targets are presented at approximately 28, 33 and 38° of eccentricity

83x90mm (300 x 300 DPI)

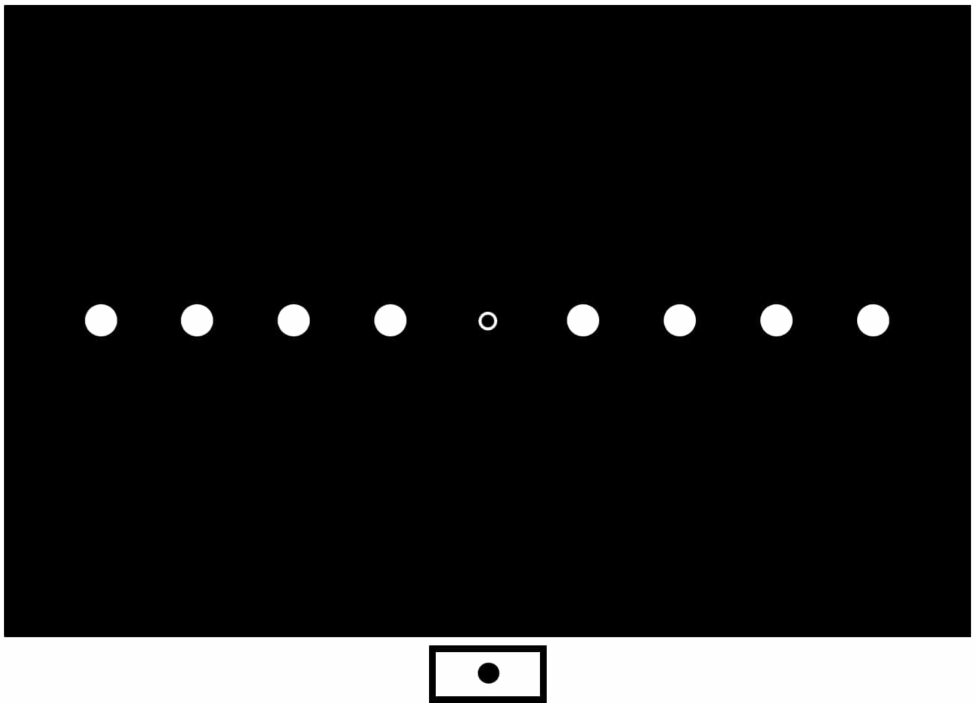

Figure 2: target positions during the radial reaching task, shown here on both the right- and left-hand sides, at 11.4, 22.6, 33.4 and 43.6° from fixation. The start-button is positioned at the bottom of the screen 40cm away from central fixation. A webcam is placed at the point of central fixation (midpoint).

112x80mm (300 x 300 DPI)

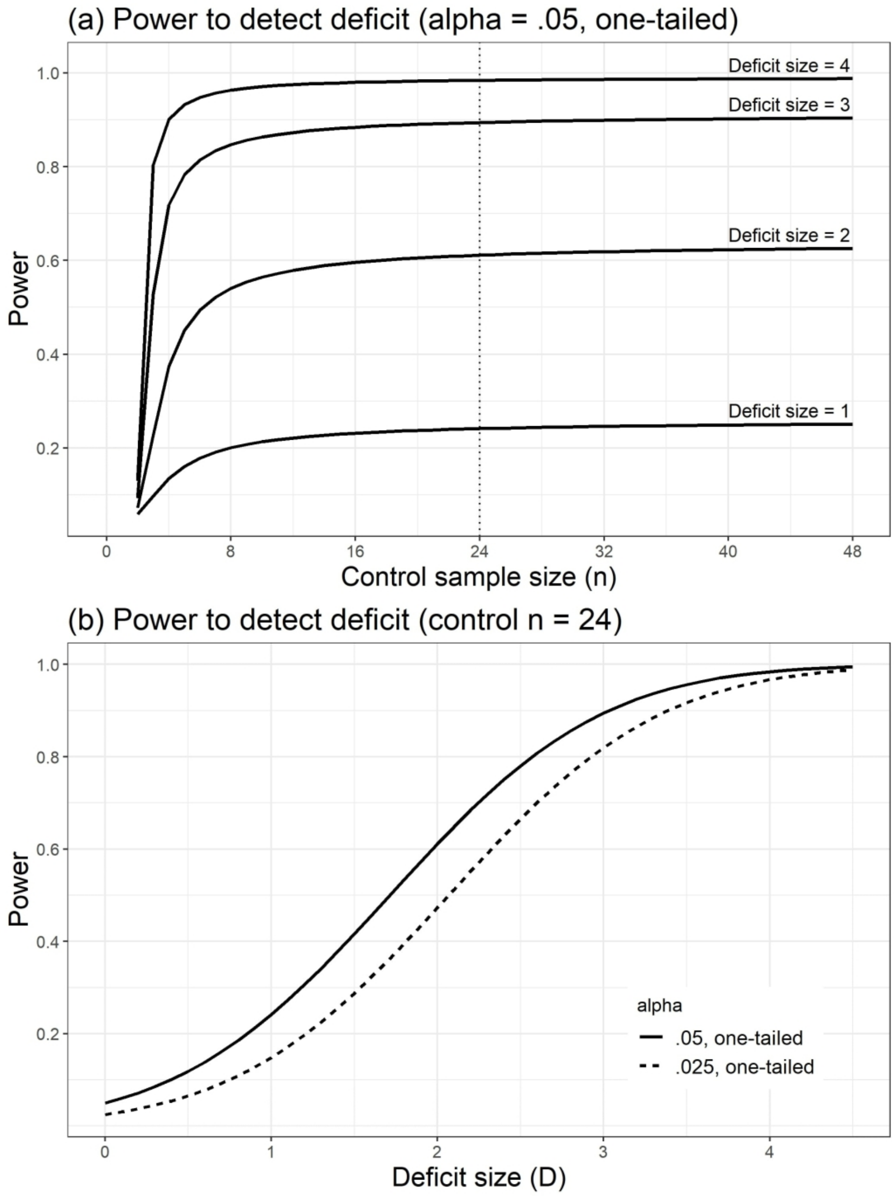

Figure 3: (a) Relation between control sample size and power to detect a single-case deficit in a one-tailed test, for different size of deficit (D, expressed as standard deviations of control mean), (b) Relation between deficit size (D) and power to detect a single case deficit, given a control sample size of 24, for adjusted and unadjusted alpha criteria.

190x254mm (300 x 300 DPI)

The assessment of visually guided misreaching in prodromal Alzheimer's disease: study protocol

Site-specific information

Site 1: Edinburgh

Patient recruitment in Edinburgh will take place at the Anne Rowling Regenerative Neurology Clinic (NHS Lothian), through a team led by Dr. Suvankar Pal. Patients who fit the recruitment criteria will be identified through the Rowling CARE-register and provided an information sheet and a notification of interest form.

All testing (patient and control) takes place in the Human Movement Laboratory, Department of Psychology, The University of Edinburgh.

Site 2: Norfolk

Patient recruitment will take place in the Julian Hospital in Norwich (NHS Norfolk & Suffolk). A team of research nurses will identify suitable participants who will be provided an information sheet and a notification of interest form.

All testing takes place in the Vision and Action Laboratory, Department of Psychology, The University of East Anglia.

For peer review only

STROBE Statement—checklist of items that should be included in reports of observational studies

	Item No	Recommendation
**Title and abstract**	1	(*a*) Indicate the study's design with a commonly used term in the title or the abstract
		(*b*) Provide in the abstract an informative and balanced summary of what was done and what was found
**Introduction**		
Background/rationale	2	Explain the scientific background and rationale for the investigation being reported
Objectives	3	State specific objectives, including any prespecified hypotheses
**Methods**		
Study design	4	Present key elements of study design early in the paper
Setting	5	Describe the setting, locations, and relevant dates, including periods of recruitment, exposure, follow-up, and data collection
Participants	6	(*a*) *Cohort study*—Give the eligibility criteria, and the sources and methods of selection of participants. Describe methods of follow-up<br>*Case-control study*—Give the eligibility criteria, and the sources and methods of case ascertainment and control selection. Give the rationale for the choice of cases and controls<br>*Cross-sectional study*—Give the eligibility criteria, and the sources and methods of selection of participants
		(*b*) *Cohort study*—For matched studies, give matching criteria and number of exposed and unexposed<br>*Case-control study*—For matched studies, give matching criteria and the number of controls per case
Variables	7	Clearly define all outcomes, exposures, predictors, potential confounders, and effect modifiers. Give diagnostic criteria, if applicable
Data sources/ measurement	8*	For each variable of interest, give sources of data and details of methods of assessment (measurement). Describe comparability of assessment methods if there is more than one group
Bias	9	Describe any efforts to address potential sources of bias
Study size	10	Explain how the study size was arrived at
Quantitative variables	11	Explain how quantitative variables were handled in the analyses. If applicable, describe which groupings were chosen and why
Statistical methods	12	(*a*) Describe all statistical methods, including those used to control for confounding
		(*b*) Describe any methods used to examine subgroups and interactions
		(*c*) Explain how missing data were addressed
		(*d*) *Cohort study*—If applicable, explain how loss to follow-up was addressed<br>*Case-control study*—If applicable, explain how matching of cases and controls was addressed<br>*Cross-sectional study*—If applicable, describe analytical methods taking account of sampling strategy
		(*e*) Describe any sensitivity analyses

Continued on next page

For peer review only

For peer review only - http://bmjopen.bmj.com/site/about/guidelines.xhtml

**Results**		
Participants	13*	(a) Report numbers of individuals at each stage of study—eg numbers potentially eligible, examined for eligibility, confirmed eligible, included in the study, completing follow-up, and analysed
		(b) Give reasons for non-participation at each stage
		(c) Consider use of a flow diagram
Descriptive data	14*	(a) Give characteristics of study participants (eg demographic, clinical, social) and information on exposures and potential confounders
		(b) Indicate number of participants with missing data for each variable of interest
		(c) *Cohort study*—Summarise follow-up time (eg, average and total amount)
Outcome data	15*	*Cohort study*—Report numbers of outcome events or summary measures over time
		Case-control study—Report numbers in each exposure category, or summary measures of exposure
		Cross-sectional study—Report numbers of outcome events or summary measures
Main results	16	(*a*) Give unadjusted estimates and, if applicable, confounder-adjusted estimates and their precision (eg, 95% confidence interval). Make clear which confounders were adjusted for and why they were included
		(*b*) Report category boundaries when continuous variables were categorized
		(*c*) If relevant, consider translating estimates of relative risk into absolute risk for a meaningful time period
Other analyses	17	Report other analyses done—eg analyses of subgroups and interactions, and sensitivity analyses
**Discussion**		
Key results	18	Summarise key results with reference to study objectives
Limitations	19	Discuss limitations of the study, taking into account sources of potential bias or imprecision. Discuss both direction and magnitude of any potential bias
Interpretation	20	Give a cautious overall interpretation of results considering objectives, limitations, multiplicity of analyses, results from similar studies, and other relevant evidence
Generalisability	21	Discuss the generalisability (external validity) of the study results
**Other information**		
Funding	22	Give the source of funding and the role of the funders for the present study and, if applicable, for the original study on which the present article is based

*Give information separately for cases and controls in case-control studies and, if applicable, for exposed and unexposed groups in cohort and cross-sectional studies.

Note: An Explanation and Elaboration article discusses each checklist item and gives methodological background and published examples of transparent reporting. The STROBE checklist is best used in conjunction with this article (freely available on the Web sites of PLoS Medicine at http://www.plosmedicine.org/, Annals of Internal Medicine at http://www.annals.org/, and Epidemiology at http://www.epidem.com/). Information on the STROBE Initiative is available at www.strobe-statement.org.

For peer review only - http://bmjopen.bmj.com/site/about/guidelines.xhtml

For peer review only
